# A new approach to a powered knee prosthesis: Layering powered assistance onto strictly passive prosthesis behavior

**DOI:** 10.1017/wtc.2023.14

**Published:** 2023-08-18

**Authors:** Steve C. Culver, Léo G. Vailati, David C. Morgenroth, Michael Goldfarb

**Affiliations:** 1 Department of Mechanical Engineering, Vanderbilt University, Nashville, TN, USA; 2 VA RR&D Center for Limb Loss and Mobility (CLiMB), Seattle, WA, USA; 3 Department of Rehabilitation Medicine, University of Washington, Seattle, WA, USA; 4 Department of Electrical and Computer Engineering, Vanderbilt University, Nashville, TN, USA

**Keywords:** Biomechatronics, Control, Human-robot interaction, Prosthetics, Design

## Abstract

This article describes a novel approach to the control of a powered knee prosthesis where the control system provides passive behavior for most activities and then provides powered assistance only for those activities that require them. The control approach presented here is based on the categorization of knee joint function during activities into four behaviors: resistive stance behavior, active stance behavior, ballistic swing, and non-ballistic swing. The approach is further premised on the assumption that healthy non-perturbed swing-phase is characterized by a ballistic swing motion, and therefore, a replacement of that function should be similarly ballistic. The control system utilizes a six-state finite-state machine, where each state provides different constitutive behaviors (concomitant with the four aforementioned knee behaviors) which are appropriate for a range of activities. Transitions between states and torque control within states is controlled by user motion, such that the control system provides, to the extent possible, knee torque behavior as a reaction to user motion, including for powered behaviors. The control system is demonstrated on a novel device that provides a sufficiently low impedance to enable a strictly passive ballistic swing-phase, while also providing sufficiently high torque to offer powered stance-phase knee-extension during activities such as step-over stair ascent. Experiments employing the knee and control system on an individual with transfemoral amputation are presented that compare the functionality of the power-supplemented nominally passive system with that of a conventional passive microprocessor-controlled knee prosthesis.

## Introduction

The current standard-of-care in knee prostheses are energetically passive devices, including microprocessor-controlled knees (MPKs), which provide high resistance to flexion during stance-phase, and a substantially lower resistance to motion during swing-phase (Berry, 2006). The high resistance in stance-phase enables stance-knee support during walking, and stance-knee yielding during slope and stair descent. The substantially lower resistance during swing-phase enables a “ballistic” swing-phase – a term used to describe knee motion generated by inertial coupling between the thigh and shank, in combination with a very low amount of knee resistance – which has been shown to be the mechanism by which swing-phase is typically achieved in normal walking (Mochon and McMahon, 1980).

Despite the efficacy of MPKs in providing stance and swing functionality (e.g., Bunce and Breakey, 2007; Seymour et al., 2007; Kahle et al., 2008), energetically passive prosthetic knees are unable to provide power generation, which is important for stance-phase knee-extension used in activities such as step-over-step stair ascent or during sit-to-stand transitions, and when non-ballistic swing-phase motion is appropriate (e.g., during stair ascent). In order to restore powered behaviors such as stance knee-extension and non-ballistic swing, several researchers have developed powered knee prostheses (e.g., see the reviews [Windrich et al., 2013; Pieringer et al., 2017]). Rather than strictly modulating resistance to motion, powered knee prostheses (PKs) provide both power-generative and power-dissipative torque–speed knee behaviors. PKs have been shown to offer enhanced functionality in several activities, particularly those that entail substantial active knee extension during stance-phase (e.g., Sup et al., 2010; Lawson et al., 2014; Ledoux and Goldfarb, 2017). However, the additional capabilities of PKs entail behavioral trade-offs relative to MPKs.

While passive MPKs generally achieve the span of (resistive) knee behaviors via a microprocessor-controlled modulated damper, PKs generally achieve the span of (active and resistive) knee behaviors via a microprocessor-controlled electric motor. In order to deliver biomechanical levels of torque from compact motors appropriate for leg prostheses, PKs require relatively large transmission ratios; the resulting reflected friction and/or inertia limit the ability of PKs to provide the essentially “free-swinging hinge” movement that is theoretically required for ballistic swing-phase. Some PKs have been developed with low-ratio electromechanical drives, which lowers the reflected impedance to levels that permit ballistic swing and energy regeneration (e.g., Warner et al., 2022). However, the drives on these devices have low-torque capabilities and therefore cannot provide the high-torque, high-power active and resistive stance capabilities provided by high-ratio PKs. As such, although some high-ratio PKs can be passively swung during swing-phase (e.g., Tran et al., 2022), the authors are unaware of any PK that provides both stance-phase capabilities and strictly passive ballistic swing-phase capabilities (i.e., full knee flexion across walking speeds without requiring knee power nor additional hip effort). Rather, high-ratio PKs employ some form of power-supplemented swing-phase motion (e.g., see review [Fluit et al., 2019]), rather than rely strictly on passive ballistic movement. Although such approaches can provide an effective swing-phase motion, they are less biomimetic than ballistic swing, do not guarantee coordination with thigh motion, and arguably diminish the physical ability of the user to physically influence the motion of the knee joint. Regarding the last point, in our experience, prosthesis users prefer knees that react to their movements, rather than knees that act of their own volition and require the user to react to the knee.

While high-ratio and low-ratio PKs have swing and stance phase deficiencies, respectively, a device that is able to assume a high-ratio configuration during stance-phase and a low-ratio configuration during swing-phase can overcome these deficiencies. As such, this article describes the design and control of a knee prosthesis that broadens the range of achievable knee behaviors by mechanically reconfiguring the knee with a two-speed transmission between the stance-phase and swing-phase of gait. Doing so enables the novel powered knee to achieve both the near “free swinging” passive knee behavior required to provide a ballistic swing-phase, and also the relatively high-torque behavior necessary to support both resistive and active stance-phase behaviors. The two-speed transmission additionally employs a unidirectional behavior in stance-phase, which facilitates detection of user intent to perform active stance-knee extension. Due to the range of impedances achieved by the two-speed transmission, the knee can be used as a strictly passive device during the majority of locomotion activities, such that powered behaviors are only employed when explicitly required. As such, a control structure for providing such “assist-as-needed” behavior is also described, where powered behaviors are layered upon baseline passive behaviors. The approach to powered assistance is premised on the idea that, whenever possible, the prosthesis defaults to behavior that requires it to react to user inputs, rather than act in anticipation of user motion. While this behavior is inherent when the prosthesis is controlled passively like an MPK, the idea behind this approach can be extended to active behaviors as well. Power can be used to either control the motion of the knee, or to assist the motion of the knee by “pushing” it in the direction that the user is moving it. Utilizing power to do the latter allows the user to initiate motion before the prosthesis provides powered assistance to that motion. The control system utilizes a six-state finite-state machine (FSM), where each state provides different constitutive behaviors which are appropriate for a range of activities. Transitions between states and torque control within states is controlled by user motion, such that the control system provides, to the extent possible, knee torque behavior as a reaction to user motion, including for powered behaviors. The two-speed knee and control structure are evaluated on a single subject with transfemoral amputation, and locomotion across several different activities are compared to a commercially available MPK.

## Methods

### Design and control approach

The design and control approach presented here is based on categorizing knee joint function during mobility activities into four behaviors: resistive stance behavior, active stance behavior, ballistic swing, and non-ballistic swing. The approach is further premised on the assumption that healthy non-perturbed swing-phase is characterized by a ballistic swing motion, and therefore, a replacement of that function should be similarly ballistic (i.e., which requires the knee joint to become essentially a free hinge with very low levels of modulated resistance). Finally, the approach assumes that power generation should only be used for (1) activities requiring net knee extension during stance-phase, such as stair ascent and sit-to-stand; and (2) activities requiring non-ballistic swing, such as swing-phase during stair ascent. When not providing power, the prosthesis should remain strictly passive. In other words, the device should be passive when possible, and powered only when necessary.

For the approach presented here, activities for which the prosthesis should function in either a passive or powered capacity are depicted in Table 1, which categorizes several common activities into one of the four stance and swing behaviors described above (note that this is not a comprehensive list and some activities are not tested in this manuscript). Active and resistive stance are analogs, wherein the knee joint primarily provides an extensive torque with knee motion either in extension (active stance) or flexion (resistive stance). Ballistic and non-ballistic swing describe the contribution of knee torque to the resultant motion of the lower leg. For ballistic swing, inertial torques (i.e., gravity and thigh acceleration) dominate knee motion, with the knee providing relatively low resistance to control motion; for non-ballistic swing, active knee torque overcomes inertial forces to reposition the lower leg. Note that resistive stance and ballistic swing are behaviors provided by current commercially available passive MPKs, while active stance and non-ballistic swing are not, since they require net power generation at the knee. Note that the approach taken here therefore requires that the powered prosthesis be capable of operating both as an MPK (i.e., in a strictly passive mode that spans both very low variable impedance for cadence-adaptive swing-phase, and also high torque and power dissipation for stance knee flexion in stair and slope descent), and also capable of providing power during non-ballistic swing and stance-knee extension. Various approaches could potentially be employed for the implementation of such behaviors. For the work presented here, a knee design that incorporates a two-speed transmission in combination with both passive and active motor control is employed in order to broaden the range of achievable impedances and torques. The resulting device is able to match (or exceed) the span of passive behaviors, impedances, and torques provided by current MPKs, and is also able to provide power for non-ballistic swing movements, and stance-knee extension, as subsequently shown.

**Table 1.** Generalized stance and swing behaviors of different functional activities

Functional activity	Stance-phase generalized behavior	Swing-phase generalized behavior	
Level-ground walking	Resistive	Ballistic	Resistive stance
Up-slope walking	Active	Ballistic	Knee joint resists flexion
Down-slope walking	Resistive	Ballistic	Active stance
Up-stairs walking	Active	Non-ballistic	Knee joint actively extends
Down-stairs walking	Resistive	Ballistic	Ballistic swing
Backward walking	Resistive	Non-ballistic	Inertial torques dominate knee motion
Slow walking	Resistive	Non-ballistic	
Stand-to-sit transition	Resistive	N/a	Non-ballistic swing
Sit-to-stand transition	Active	N/a	Active knee torque dominates knee motion
Stumble recovery	Resistive	Non-ballistic	
Obstacle avoidance	N/a	Non-ballistic	

### Prosthesis hardware

**Figure 1.**
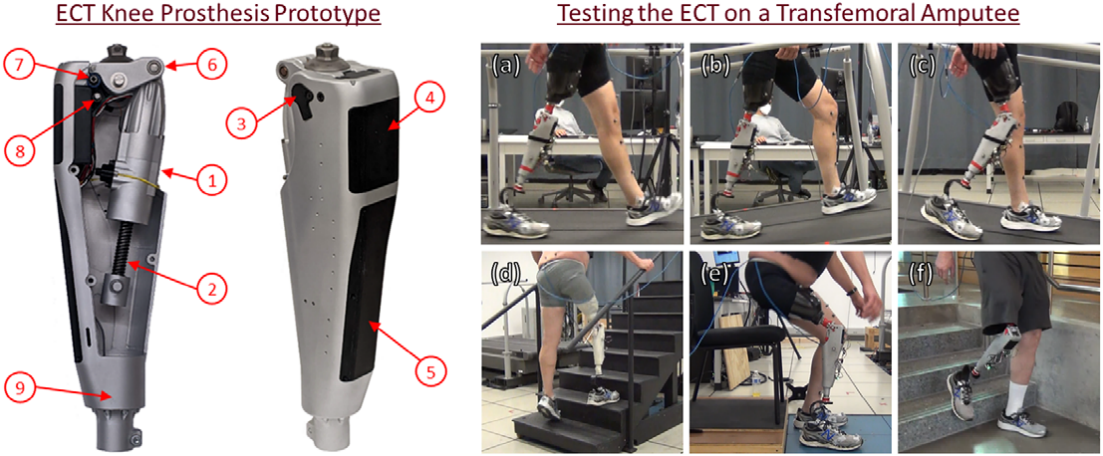
ECT knee prosthesis prototype with one half of the housing removed (left) displaying the novel actuator (1) and lead screw (2), and fully assembled prosthesis (center) including absolute encoder (3), battery pack (4), and custom embedded system (5). The range of motion of the crank arm (6) is limited by the hard stops for extension (7) and flexion (8). A load cell (9) is fixed to the bottom of the prosthesis and attaches to the pylon clamp. The ECT was evaluated on a subject with transfemoral amputation in several experiments (right): (a) level-ground, (b) up-slope, and (c) down-slope walking on an instrumented treadmill; (d) upstairs and downstairs walking; (e) sit-to-stand and stand-to-sit transitions; and (f) an overground walking circuit that incorporates all aforementioned activities and the transitions between them.

The control approach described here is implemented on a knee prosthesis prototype called the ECT knee, described in detail in Culver et al. (2022) and shown in Figure 1. The prosthesis is actuated using a DC brushless motor (Maxon EC-4pole 22 90 W), which is connected to the knee joint through a novel two-speed electronically controlled transmission (ECT). The motor is controlled either passively or actively, depending on the control mode (i.e., passive for resistive stance and ballistic swing, active for active stance and non-ballistic swing). When controlled passively, the motor leads are shorted via a PWM control signal, such that the battery cannot provide power to the motor. Even though the battery cannot power the motor in this passive mode of control, the motor is connected to the battery through a set of diodes, such that the motor can regenerate power to the battery during passive operation. This passive motor control approach is described in detail in Vailati and Goldfarb (2022). When controlled actively, the motor driver employs a standard current control technique with block commutation. The two-speed transmission of the knee provides two functional regimes of operation for the prosthesis: (1) a highly backdrivable low-gear that enables ballistic swing-phase motion in passive control mode or non-ballistic swing in active control mode, and (2) a relatively higher gear that enables high-torque stance-knee flexion in passive control mode and powered stance-knee extension in powered control mode. The ECT is based on an underdetermined planetary gear transmission that is configured (i.e., made determinate) via a pair of clutches, one unidirectional, one bidirectional, which can be engaged or disengaged in various combinations to provide: (1) a high-gear ratio appropriate for stance-phase; (2) a low-gear ratio appropriate for swing-phase; and (3) a combination that enables a low-gear ratio against extension and locks against flexion. The unidirectional clutch ensures that the knee always allows stance-knee extension during stance phases of gait.

**Figure 2.**
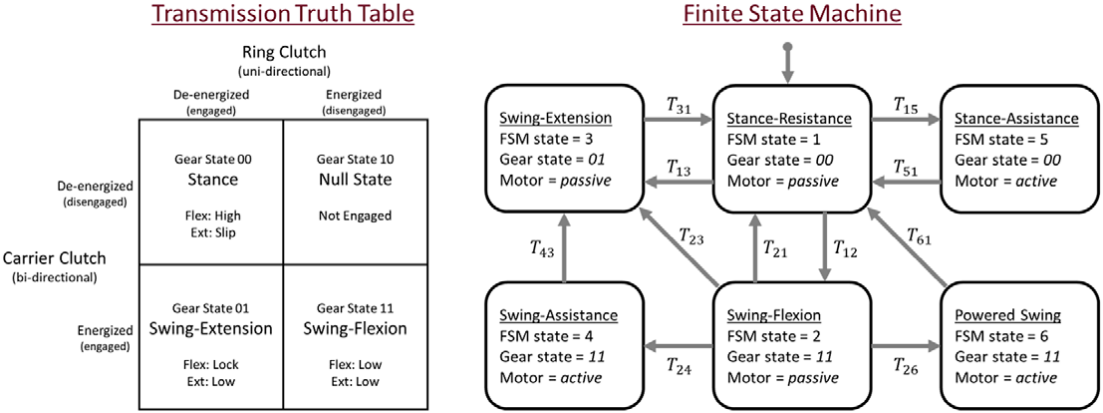
The ECT is an under-determined transmission with two clutches that ground different components and provide different reduction ratios between input and output (for more details about the mechanical design, see Culver et al., 2022). Although the ECT has two defined gear ratios, there are four possible mechanical states of the transmission (two clutches each with two states). The transmission truth table illustrates how engaging and disengaging the ring and carrier clutches produces different functional states in flexion and extension: a low transmission ratio (low), a high transmission ratio (high), over-determined locking (lock), and under-determined motion between input and output (slip or not engaged). Functionality of the prosthesis in gear states 00, 11, and 01 is demonstrated in the Supplementary Video. The FSM for walking consists of six states and the transitions between them. The FSM operates by controlling the output torque and impedance via hardware configuration and motor torque commands. Within each state, the motor is commanded to operate passively (i.e., constrained to braking behavior) or actively (i.e., using a standard block-commutation scheme). The FSM diagram indicates the corresponding gear states of the hardware and if the motor is operated actively or strictly passively. Torque control laws within each state are outlined in Table 2; transition conditions between states are outlined in Table 3.

The transmission ratios between the low and high gears are different by a factor of 5.4, which combined with passive motor control enables two orders of magnitude variation in passive impedance, and also renders it capable of providing an appropriate range of torques for powered stance and swing behaviors. Although there are two defined gear ratios, there are four possible mechanical states of the transmission, since the two clutches each have two states. These four transmission states are outlined on the transmission truth table in Figure 2 and described below:

*Gear state 00* – Both clutches are de-energized. The ring-gear is locked and the transmission provides a high-ratio extension torque, but due to the unidirectional nature of the ring clutch, provides no flexion torque. This is the stance-resistance and stance-assistance gear state.

*Gear state 10* – The carrier clutch is de-energized and the ring clutch is energized. The transmission assumes an under-determined state, which effectively disengages the output shaft from the drive motor. This state is assumed for several milliseconds in order to prevent over-constraining the transmission against flexion before transitioning into the swing-flexion state.

*Gear state 11* – Both clutches are energized. The first-stage carrier is locked and the ring-gear is unlocked. The transmission provides a low-ratio in both directions. This is the swing-flexion state.

*Gear state 01* – The carrier clutch is energized and the ring clutch is de-energized. The unidirectional nature of the ring clutch allows the ring-gear to slip against the clutch in the extension direction. The transmission is in low-gear when driven in the extension direction, but locks when driven in the flexion direction. This is the swing-extension state. The ECT enters this state in order to preconfigure the prosthesis for the stance-phase in case there is a sudden need to load the prosthesis (e.g., due to stumble).

### Embedded system

A custom embedded system was designed for sensing, actuation, and control of the prosthesis. The embedded system includes: (1) power electronics to drive a brushless motor and the two clutch solenoids; (2) sensing and signal conditioning, including current sensing for motor and solenoids, encoders at the motor shaft and knee joint for measurement of knee joint position and velocity, shank axial force load measurement, and a six-axis inertial measurement unit for measurement of the shank and thigh angles and velocities; (3) two microcontrollers which implement various control tasks; and (4) controller area network (CAN) communication hardware. The system is powered using four 18650 batteries in series, providing nominal 16.8 V with a maximum continuous current capacity of 15 A. The CAN interface is used to exchange control data (i.e., sensor values and control parameters) at 500 Hz with a computer which runs a high-level controller on MathWorks Simulink Desktop Real-Time. For the passive mode of motor control, the three low-side MOSFETs of the bridge are switched simultaneously, which guarantees smooth and strictly passive braking behavior; in the active mode of motor control, a standard block-commutation scheme is used to provide current control in either active or passive behavioral regimes, as determined by the activity control system.

### Activity control system

The activity controller is a six-state FSM, diagrammed in Figure 2, which provides full passive and powered functionality across a wide range of activities. Each state within the FSM has a unique transmission configuration and a unique torque control law (see Table 2), providing either power dissipation (passive motor control) or generalized active and emulated-passive behaviors (active motor control). Passive and active control methods, as well as low-level motor control is discussed in detail in Culver et al. (2022). Within each state, the knee torque is governed by a torque control law based on sensor inputs and control parameters that produce the following behaviors in each corresponding FSM state: (1) high-torque turbulent damping; (2) low-torque cadence-adaptive viscous damping; (3) low-torque unidirectional cadence-adaptive viscous damping that increases as the knee approaches full extension; (4) low-torque cadence-adaptive flexion torque pulse; (5) high extension torque that scales with residual hip torque and velocity; (6) low-torque PD controller with a virtual linkage between the thigh and knee joint, adapted from Lee and Goldfarb (2021). FSM transitions, shown in Table 3, are governed by onboard sensing of knee angle ( 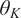 ), shank angle ( 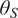 ), shank axial force ( 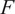 ), shank axial acceleration ( 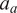 ), the walking speed estimation ( 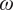 ), and a state timer ( 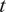 ). Depending on the activity, the FSM transitions produce different FSM sequences. Table 4 shows the FSM sequences for different activities. Note that the FSM provides appropriate resistive or active stance-phase behavior and appropriate ballistic or non-ballistic swing-phase behavior, depending on the activity. Regarding tunability of parameters, it is not necessary to tune any of the transition condition threshold parameters in Table 3, and it is only necessary to tune some of the torque command parameters in Table 2 (e.g., individual users will not require tuning of 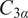 , 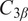 , 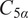 , or 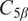 ).

**Table 2.** Finite state torque control laws

FSM state		Torque control law
1	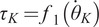	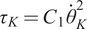
2	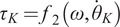	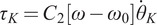
3	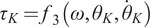	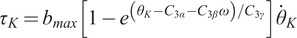
4	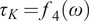	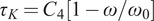
5	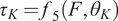	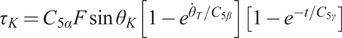
6	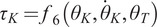	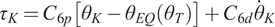

**Table 3.** Finite-state machine transition conditions

Transition	Description	Condition
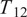	Knee joint is hyperextended, and	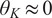
	Prosthesis shank is rotating forward, and	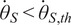
	Prosthesis shank is inclined forward, and	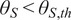
	Prosthesis is rapidly unloaded	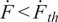
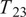	Prosthesis is unloaded, and	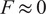
	Knee joint is extending	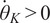
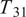	Knee joint has zero velocity, or	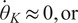
	Prosthesis is loaded	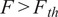
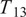	Prosthesis is unloaded, and	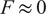
	Knee joint is flexed past threshold	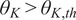
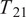	Prosthesis is loaded, and	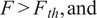
	Prosthesis was previously unloaded, or	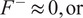
	Prosthesis shank rotating backward, or	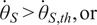
	Prosthesis shank is not inclined forward	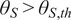
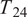	Knee joint begins flexing, and	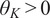
	Slow walking speed detected, and	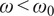
	Walking speed above threshold	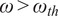
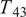	Prosthesis is unloaded, and	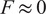
	Knee joint is extending	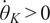
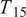	Knee joint is flexed past threshold, and	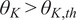
	Knee joint is extending	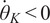
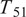	Knee joint is fully extended, or	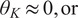
	Knee joint is flexing	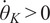
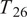	Knee joint is hyperextended, and	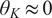
	Prosthesis is unloaded, and	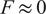
	Shank axial acceleration above threshold	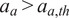
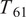	Prosthesis is loaded, or	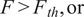
	Time in state beyond threshold	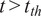

Finite-state machine transition conditions depend upon measured sensor inputs (see caption for Tables 2 and 3) and several threshold parameters: knee angle ( 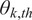 ), shank angle ( 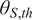 ) and angular velocity ( 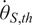 ), shank axial force ( 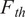 ) and yank ( 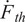 ), walking speed estimation ( 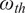 ), shank axial acceleration ( 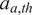 ), and time ( 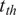 ).

**Table 4.** Controller sequence for different activities

FSM sequence	Functional activities
1	Standing; stand-to-sit; backward walking
1–2–3	Level-ground and up-slope walking
1–2–4–3	Slow walking (level-ground and up-slope)
1–3	Down-slope and down-stair walking
1–5	Sit-to-stand
1–2–6–1–5	Up-stairs walking

Within each FSM state, knee torque is governed by a unique control law based upon a combination of sensor inputs: knee angle ( 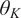 ) and velocity ( 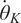 ), thigh angle ( 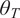 ), shank axial force ( 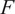 ), and walking speed estimation ( 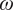 ). Each torque control law *(* 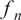 ) has between one and three tunable parameters ( 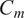 ). For 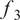 , 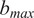 is a system parameter that indicates the maximum achievable motor braking impedance (i.e., when the motor leads are connected at 100% duty cycle). For 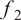 and 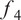 , 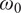 is a tunable parameter that indicates the crossover walking speed, which is the walking speed where the motor provides neither assistance nor resistance and swing-flexion motion is governed by passive dynamics alone. For 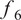 , 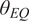 is the equilibrium position of the virtual spring, which is a function of the thigh kinematics, as described in Lee and Goldfarb (2021). For 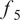 , 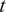 is time as relevant for the filter term.

#### Resistive and active stance

**Figure 3.**
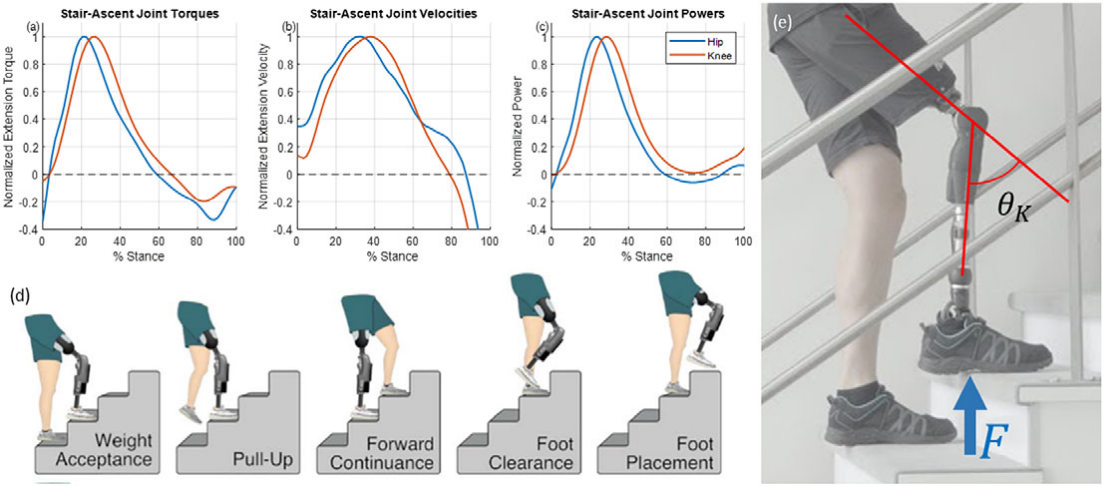
Stance-phase (a) torque, (b) velocity, and (c) power of knee and hip joints during the stance-phase of stair ascent, from Riener et al. (2002). Positive *y*-axes indicate (a) extension torque, (b) extension velocity, and (c) power generation. Values are normalized to a maximum of unity. These plots demonstrate that the phasing of the knee joint lags the hip joint for most of the stance-phase. (d) The phases of stair-ascent as described in McFadyen and Winter (1988), image adapted from Lee and Goldfarb (2021). (e) When ascending stairs with a stiff prosthetic foot, the shank is constrained to be approximately vertical. The hip torque can be approximated by onboard sensors as the product of the load cell force (*F*) and the sine of the knee angle ( 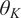 ). The control system uses this approximation of hip torque to deliver knee torque that is synchronized with the user’s motion.

FSM states 1 and 5 provide the necessary mechanical power dissipation and generation during stance-phase to accomplish a variety of functional activities. Turbulent damping via passive motor control provides knee-yielding akin to a commercial MPK, to provide appropriate knee motion during down-slope and down-stairs walking, as well as stand-to-sit (Wolf et al., 2012). The novel active stance control law, which uses the motor in active mode, generalizes powered knee extension into a single torque control law that is adaptive across a range of activities that benefit from positive joint power. The control law was developed from observations of the interaction between the biological knee and hip joints during stair ascent. As shown in Figure 3, the torque, velocity, and power of the biological knee joint lag behind those of the hip joint during stair ascent. The torque command in Table 2 was designed to input force and motion estimates of the residual hip joint and reproduce the shape and timing of the torque profile of the biological knee joint, but without commanding a desired joint angle, which would otherwise have the prosthesis, rather than the user, control knee motion. During the pull-up phase of stair ascent, the prosthetic ankle constrains the shank to be approximately vertical; as such, the real-time hip torque is estimated as the product of the load cell force and the sine of the knee angle (see Figure 3e). The thigh velocity and knee angle terms in the control law (see Table 2) provide for the bell shape of the torque command, and the filtering term provides the phase delay between hip and knee kinetics and kinematics. Ideally, the parameters 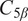 and 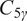 are invariant between users, providing the torque control law a single parameter ( 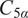 ) that increases or decreases the magnitude of assistive torque, depending on the user’s preference.

The unidirectional high-gear of the ECT enables the prosthesis to provide an extension torque that either passively resists flexion or actively provides extension. Additionally, due to the unidirectional clutch, there is little resistance to user-initiated stance knee-extension (i.e., the user can extend the stance leg via hip musculature without drivetrain resistance). Because the prosthetic foot is frictionally constrained to the ground during stance-phase, the stance leg is a closed kinematic chain, and therefore, the prosthesis user has control of knee joint movement during the stance-phase via movement of the hip joint. The user is therefore able to extend the knee joint without power-assistance, albeit with disproportionate hip torque input from the user, as demonstrated in Lee and Goldfarb (2021). In the device described here, powered knee extension is activated by the user via hip torque, which initiates a knee extension movement, which in turn is identified by the controller and used to initiate power-assisted knee extension. Because the user is able to volitionally control the activation of powered stance knee-extension, intent recognition algorithms are not necessary for coordinated control; the coordination is inherent because power delivery is solely in reaction to the motion input generated by the user. Additionally, the thigh velocity term in the torque control equation scales torque delivery with estimated thigh power. Just as the biological hip and knee work synergistically to extend the leg when it is in a closed kinetic chain, the prosthetic knee is able to follow motion and force cues from the residual hip (under the user’s neuromuscular control) and provide synchronous assistive knee torque. In this manner, the user does not ride the prosthetic knee up the stairs, but rather works with it to extend the leg, similar to the manner in which an electric bicycle coordinates its power delivery with the user’s power input. While it is possible to cause controller instability using such a method, since a velocity term is used in the torque control law to add energy, instability is avoided by the combination of making the control law unidirectional, using an exponential decay as a soft saturation on the velocity term, and using the sine of the knee angle to decay the torque as the knee extends. With this control law formulation, if the user stops extending their hip, the user’s mass decelerates the knee joint, which reduces the torque and continues the deceleration. When knee velocity inflects, the FSM switches to resistive stance behavior, providing controlled support of the user’s weight as the knee flexes.

#### Ballistic swing

**Figure 4.**
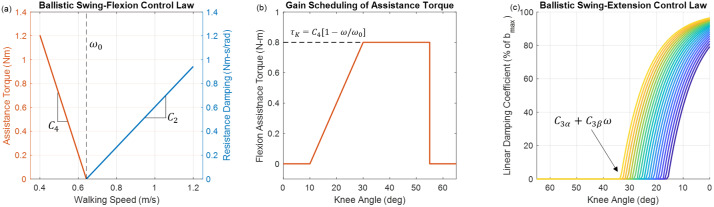
Ballistic swing torque control laws, incorporating both assistive and resistive behaviors. (a) During swing-flexion, for walking speeds below 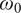 , an assistive torque is provided; for walking speeds above 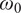 , a resistive torque is provided. Both torque control laws are linearly proportional to walking speed, which provides cadence-adaptive behavior. (b) Gain scheduling of swing-flexion assistance torque. After the user has flexed the knee joint past 10°, the assistance torque begins ramping up to the commanded value as a function of knee angle. At 30° of flexion, the assistance torque has reached its commanded value. After 55° of flexion, the commanded torque is zeroed so the knee joint velocity can inflect for swing-extension. (c) The swing-extension torque control law commands a linear damping torque where the linear damping coefficient is a function of the walking speed and the knee angle (walking speeds from slow to fast are graded from blue to yellow). The commanded torque is zero until a predetermined angle that is a function of the walking speed estimation ( 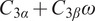 ). After this point, the damping coefficient rapidly increases toward the maximum value ( 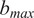 ). An exponential curve serves as a soft saturation of knee damping.

FSM states 2–4 provide walking-speed-adaptive ballistic swing phase behavior (see Figure 4). To estimate walking speed, the shank angular velocity is recorded and averaged from foot contact until the user initiates swing-phase in late-stance. The result is a linear relationship between the value of the average stance-phase shank angular velocity ( 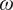 ) and the walking speed. As such, 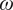 is a zero-parameter term that measures relative changes in walking speed within a single stride and can be used directly in control equations to provide cadence-adaptive behavior. During swing-flexion, when estimated walking speeds are above the crossover walking speed (i.e., when 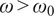 ), a damping torque is provided, similar to a commercial MPK. When 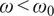 , a feedforward assistive flexion torque is provided, which increases the peak-flexion knee angle to a biomimetic value not achievable with passive dynamics alone. This assistive torque has low amplitude and is provided unidirectionally, without trying to control knee angle directly, which enables a swing-phase motion that is still inertially coupled (i.e., ballistic swing is preserved because low actuator impedance makes the knee joint receptive to inputs from inertial forces), but with the caveat that the motor is helping the user by “pushing” the lower leg toward flexion.

FSM state 3 provides cadence-adaptive ballistic swing-extension behavior (see Figure 4c), which is appropriate for the swing-extension phase of all evaluated walking activities except for stair ascent, which requires non-ballistic swing. For level and up-slope walking, swing-extension behavior is provided immediately after peak-flexion; for down-slope and down-stairs walking, swing-extension behavior is provided when the user lifts the flexed prosthetic knee, allowing inertial and gravitational forces to provide the extensive torque.

#### Non-ballistic swing

FSM state 6 provides non-ballistic swing-phase motion appropriate for stair-ascent. The state 6 controller was adapted from Lee and Goldfarb (2021), albeit with a modification that the FSM must first pass through state 2, ensuring that powered swing is provided as a transition from late-stance similar to other walking conditions. This controller creates a virtual linkage between the thigh and shank, which enables the user to volitionally control the knee joint. To contrast ballistic and non-ballistic swing controllers, the former is controlled through inertial coupling while the latter is controlled through kinematic coupling. Similar control methods have been used by other researchers for obstacle crossing (e.g., Mendez et al., 2020).

### Experimental assessment

An experimental assessment was performed to investigate: (1) the ability of the prosthesis and control system to provide strictly passive functionality essentially identical to a state-of-the-art daily-use MPK; (2) the ability of the prosthesis to provide powered functionality for non-ballistic swing and stance-knee extension; and (3) the ability of the control system to seamlessly transition between the aforementioned activities. The experimental assessments consisted of four tests (see Figure 1): (1) treadmill walking on level-ground and ramps; (2) up-stairs and down-stairs walking; (3) sit-to-stand and stand-to-sit transitions; and (4) walking in an over-ground circuit with level-ground, ramps, stairs, and sitting/standing. The assessments were conducted on a single subject with transfemoral amputation – a 62-year-old male with traumatic amputation (50 years ago), K-4 activity level, weighing 85 kg, who used an Ottobock C-Leg 4 as his daily-use prosthesis (8 years with C-Leg). The subject completed 100 hr of testing on the prosthesis throughout the development of the control systems. In experiments 1–3, the subject first conducted the protocol wearing his daily-use MPK, then followed the same protocol wearing the ECT knee, using the same socket and prosthetic foot (a Fillauer Allpro). The ECT knee was neutrally aligned in a similar manner to his C-Leg 4 by the user’s prosthetist. Total masses of the ECT prosthesis and subject’s MPK, including prosthetic foot, shell, and shoe, were 3.50 kg and 3.25 kg, respectively. Kinematic data were recorded via a motion capture system (Vicon), and ground reaction forces were recorded via force plates integrated into either a Bertec instrumented treadmill for experiment 1, or into the floor (AMTI) for experiment 3. Data used by the embedded system to implement the control tasks was also collected and is presented when appropriate.

Experiment 1 had three treadmill walking conditions: (1) level walking at nine treadmill speeds between 0.4 and 1.2 m/s; (2) upslope walking at a slope of 8° at three treadmill speeds between 0.5 and 0.7 m/s; and (3) downslope walking at a slope of −8° at three treadmill speeds between 0.5 and 0.7 m/s. In the level-ground walking trial, speeds were ramped up from 0.4 to 1.2 m/s, incrementing by 0.2 m/s, then speeds were ramped down from 1.1 to 0.5 m/s, decrementing by 0.2 m/s. The subject was allowed to reach steady-state before motion capture data were recorded, and 15 strides of steady-state walking were recorded for each walking speed. In the up-slope and down-slope trials, speeds were ramped up from 0.5 to 0.7 m/s at 0.1 m/s increments, and data were recorded in the same manner as level-ground trials. The expectation of experiment 1 is that the ECT knee will demonstrate the same resistive stance and ballistic swing-phase behaviors as the C-Leg with improved swing-flexion kinematics at slower walking speeds (due to swing-assistance).

Experiment 2 involved ascending and descending an eight-step staircase five times using a step-over gait. Each step was 17 cm (6.5 in) high. The subject was considered to be in steady-state on each step except for the first and last steps, resulting in a total of 15 strides per device. The subject was allowed to use the staircase handrails for balance. The expectation of experiment 2 is that the ECT knee will have a knee trajectory more similar to the contralateral knee than the C-Leg, as previously demonstrated in comparisons of powered knee prostheses to MPKs (Lawson et al., 2013).

Experiment 3 involved standing from and sitting to a chair 10 times with each foot placed on separate force plates. The subject did not use his arms to aid standing as to not compromise inverse dynamics. For experiments 1–3, the subject rested 5 min between trials, and all data were recorded on the subject’s daily-use MPK before the subject was fit with the ECT knee, and repeated the same experimental procedures. The expectation of experiment 3 is that the ECT knee will show significant increase in prosthetic side joint power and ground reaction force with a simultaneous decrease in intact side joint power and ground reaction force, relative to the C-Leg, as previously demonstrated in comparisons of powered knee prostheses to MPKs (Wolf et al., 2012).

In experiment 4, the subject completed a single loop through a circuit that included level-ground, turns, ramps, stairs, and sitting/standing with a chair. Knee angle and controller state data were recorded using the embedded system. This circuit was only completed once to demonstrate the ability of the control system to adapt to a variety of ambulation conditions, rather than to evaluate the biomechanical efficacy of the controller (which is demonstrated with experiments 1–3). The expectation of experiment 4 is that the subject will be able to complete all tasks with biomimetic movement and without hesitation when transitioning between activities. The experimental protocol was approved by the Vanderbilt Institutional Review Board. The experimental participant provided written informed consent before his participation as required by the approved protocol.

## Results

**Figure 5.**
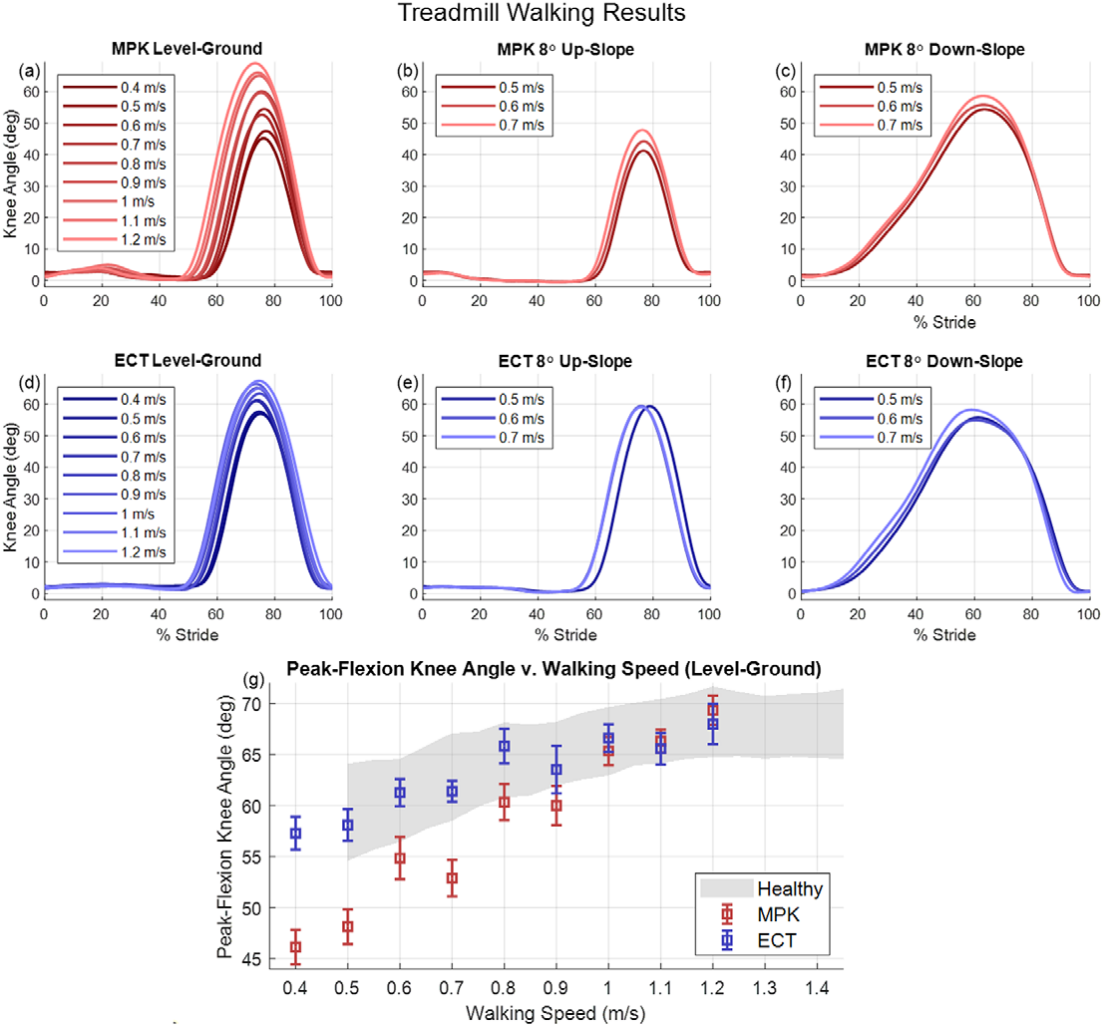
Experimental results showing 15-stride average knee angle of (a) commercial MPK and (d) ECT in level-ground walking at walking speeds between 0.4 to 1.2 m/s; (b) commercial MPK and (e) ECT in up-slope walking on an 8-degree ramp at walking speeds between 0.5 to 0.7 m/s; and (c) commercial MPK and (f) ECT in down-slope walking on an 8-degree ramp at walking speeds between 0.5 to 0.7 m/s. (g) Peak-flexion knee angle vs. walking speed during level-ground walking, comparing ECT (blue) to a commercial MPK (red) and control data from healthy subjects (shaded gray) (Camargo et al., 2021). Box-and-whisker plots indicate 15-stride mean and standard deviation of the peak swing-flexion knee angle for each walking speed in level-ground walking. Shaded gray areas indicate range of 1 standard deviation of averaged maximum knee angle data from 28 healthy subjects (Camargo et al., 2021). In plot (g), both prostheses show a trend of increasing peak-flexion knee angle with increasing walking speed. The MPK deviates from healthy data at walking speeds below 0.8 m/s.

**Figure 6.**
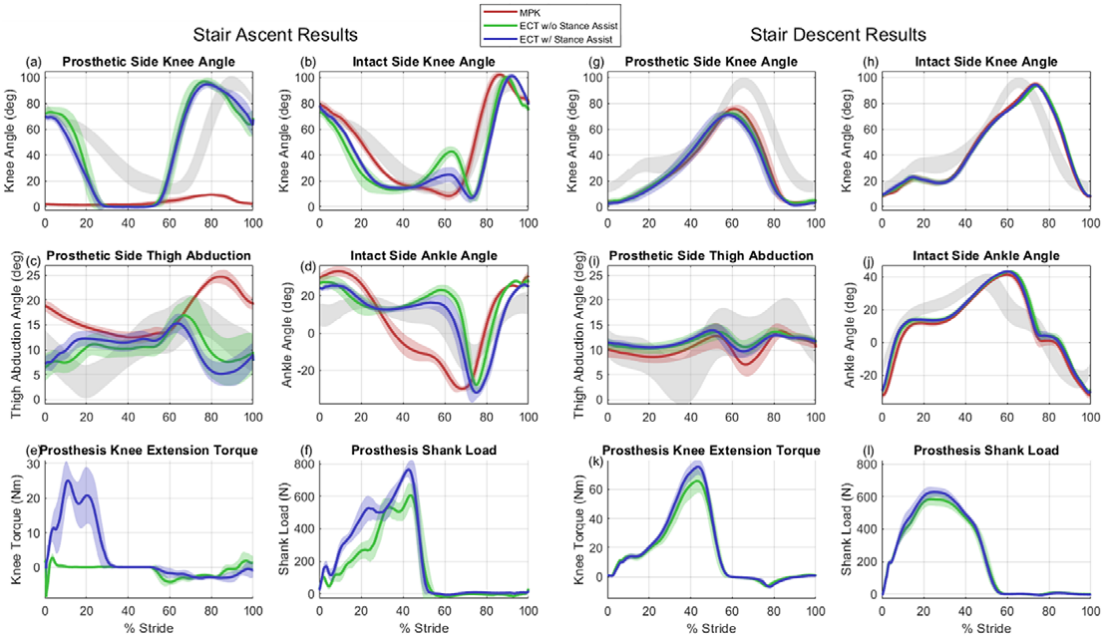
Averaged stair ascent and descent data, showing (a,g) prosthetic side knee angle, (b,h) intact side knee angle, (c,i) prosthetic side thigh abduction angle, (d,j) intact side ankle angle (positive direction is dorsiflexion), (e,k) prosthesis knee extension torque (from embedded system data and actuator model), and (f,l) axial load on prosthetic shank. Most plots compare three prosthesis conditions: subject’s daily-use MPK (red), ECT without stance-assistance (green), and ECT with stance-assistance (blue); plots (e,f,k,l) only compare the latter two conditions, since shank load is recorded by the ECT’s load cell only and knee torque is calculated from the motor current and actuator friction model. Shaded regions on plots (a–d) and (g–j) indicate range of 1 standard deviation of averaged data from 28 healthy subjects (Camargo et al., 2021).

**Figure 7.**
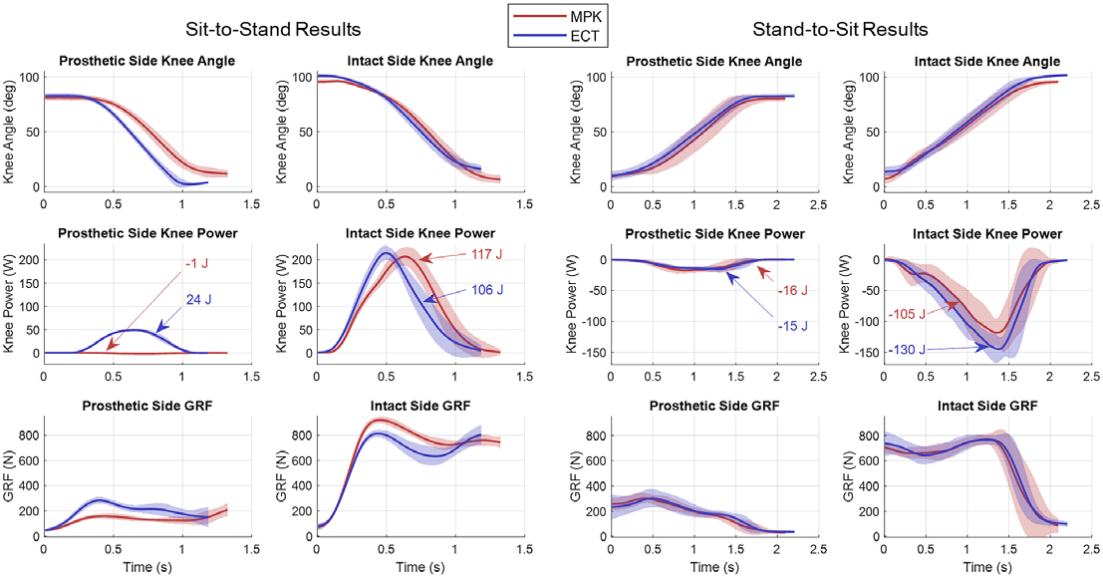
Averaged sit-to-stand and stand-to-sit data, showing knee angle, knee power, and ground reaction force (GRF) on both the prosthetic and intact sides. Each plot compares two prosthesis conditions: subject’s daily-use MPK (red), and ECT with stance-assistance (blue). Knee power plots show the total energy generated or dissipated by each side when standing up or sitting down.

**Figure 8.**
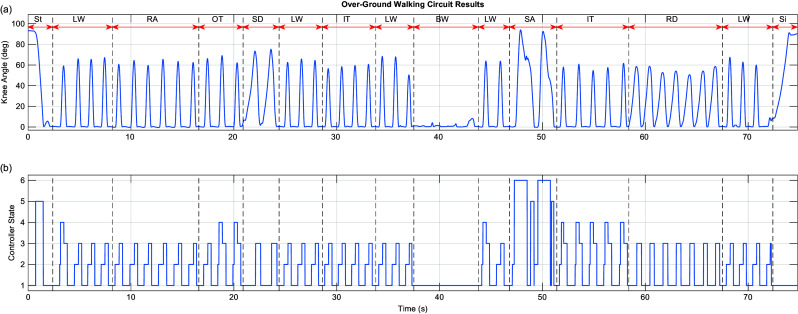
Results of over-ground walking circuit showing (a) knee angle and (b) FSM controller state for the entire circuit. For LW, RA, IT, and OT, FSM state-flow is 1–2–3. For slow walking, FSM state flow is 1–2–4–3. For RD and SD, FSM state-flow is 1–3. For SA, FSM state-flow is 1–2–6–1–5. Activity abbreviations: LW, level; BW, backward walking; RA/RD, ramp ascent/descent; SA/SD, stair ascent/descent; IT/OT, inside/outside turn; St, standing; Si, sitting. FSM controller states as presented in Figure 2: 1, stance; 2, swing-flexion; 3, swing-extension; 4, swing-assistance; 5, stance-assistance; 6, powered swing.

This section is organized into four subsections that correspond to the four experiments conducted: (1) treadmill walking on level and sloped ground, (2) stair ascent and descent, (3) sit-to-stand and stand-to-sit transitions, and (4) overground walking with multiple activities. Figures 5–7 show data corresponding to experiments 1–3 with both the subject’s daily-use MPK and the ECT. Specifically, Figure 5 shows the mean knee angle over 15 strides as a function of stride, and corresponding peak-flexion knee angle of peak-flexion as a function of walking speed. Figure 6 shows the 15-stride mean values of prosthetic and intact side knee angle, prosthetic side thigh abduction, intact side ankle angle, knee torque, and shank axial load as a function of stride for stair ambulation. Figure 7 shows the mean values across 10 sit-to-stand trials of knee angle, knee power, and ground reaction force as a function of time for both the intact and prosthetic sides. Figure 8 shows data corresponding to experiment 4, showing knee angle and controller state for the ECT throughout the walking circuit. Videos corresponding to these experiments are included with the Supplementary Material submitted with this article.

### Treadmill walking on level and sloped ground

During level-ground, up-slope, and down-slope treadmill walking, the stance and swing phase kinematics are highly similar between the ECT and MPK across activities and speeds, which was the design goal. Figure 5a–f shows the knee angle of the ECT and MPK as a function of stride for a range of walking speeds during level-ground, up-slope, and down-slope walking. For level-ground and up-slope walking on both prosthetic knees, the knee joint remained extended during the stance-phase and flexed to an angle between 40° and 70° in the swing-phase, depending on walking speed. The swing-phase trajectory of each prosthetic knee has a bell shape of similar duration for each walking speed and slope. Figure 5g shows the peak-flexion knee angle of both prostheses across walking speeds during level-ground walking, along with corresponding data from healthy subjects. As shown in the figure, between 0.8 and 1.2 m/s, the peak-flexion knee angles were similar between prosthetic knees; at walking speeds below 0.8 m/s, however, the peak-flexion knee angles of the ECT more closely matched the healthy data, relative to the MPK.

For up-slope walking, the ECT exhibited higher peak-flexion knee angles than the MPK, closer to the values in level-ground walking for each walking speed. From healthy subject data, the maximum angle of the biological knee in swing-phase is approximately the same as level-ground walking across different slope ascent angles (Camargo et al., 2021). In both cases (slow level-ground and slow up-slope walking), swing-assistance torque increased the peak-flexion knee angle to biomimetic levels, which would otherwise not be achievable using passive dynamics alone. During down-slope walking, both prosthetic knees showed similar knee trajectories for stance and swing phases. During the stance-phase, the knee joint yielded from heel-strike to toe-off, supporting the subject’s weight as the center-of-mass progresses forward and downward. The knee joint flexed to approximately 55° to 60° during stance-phase for all walking speeds for both prosthetic knees. The subject initiated swing-phase by swinging the prosthetic thigh forward, lifting the prosthetic foot from the ground. Inertial coupling from thigh acceleration and gravity caused the knee joint to extend. For downslope walking with both prosthetic knees, the swing-phase had approximately the same spatial–temporal characteristics across all walking speeds.

### Stair ascent and descent

The subject ascended and descended stairs (with the use of handrails) in a step-over-step manner using both prosthetic knees. As shown in Figure 6g–l, there were no notable differences between prosthetic knees when descending stairs, which was the expected result since the ECT emulates the same turbulent damping as the MPK during resistive stance. During stair ascent, the ECT knee’s addition of non-ballistic swing and active stance functionality produced a knee trajectory more similar to the contralateral knee than the MPK, which is the expected result from previous studies of powered knees during stair ascent (Lawson et al., 2013). As shown in Figure 6a, during stair ascent the subject was unable to elicit swing-flexion with the MPK, since the kinematics of stair ascent are not amenable to ballistic swing (i.e., the subject would swing the foot into the stair riser). Instead, the subject employed hip circumduction, as indicated by significant thigh abduction in Figure 6c, with an essentially extended knee in order to ascend stairs in a step-over fashion, similar to the observations reported in Lee and Goldfarb (2021). In contrast, the ECT provides a powered swing function (FSM state 6), which produced essentially biomimetic swing kinematics during stair ascent. Figure 6e,f shows the knee torque and shank loading for the two cases of ECT stair ascent – one with stance assistance, and one without, showing the increased prosthesis loading during stance-phase when the motor provides stance knee-extension assistance, indicating that the subject is not fully loading the prosthesis until the knee joint is fully extended when there is no assistance. This reduced loading on the prosthetic side must therefore be offloaded to the intact side and/or the handrails. When not providing stance-assistance, the subject vaulted over the prosthetic leg, simultaneously pulling with his arms while extending his hip to straighten the knee joint. As such, the knee joint extended more quickly without stance knee assistance relative to the case with stance assistance, as indicated by the difference in the rate of change of knee angle during early-stance in Figure 6a.

The subject exhibited compensatory behavior in all three cases while ascending stairs. Figure 6b,d shows that, similar to Hobara et al. (2011), the subject’s intact side knee and ankle joints showed rapid flexion and subsequent extension in late-stance for the two ECT cases. This is likely a compensatory strategy to increase center of mass velocity during the pull-up phase with the prosthetic side. This compensatory motion is more pronounced when not providing any assistive torque, which indicates: (1) a relatively small amount of assistive torque (~25 N-m) is sufficient to reduce compensatory intact side knee and ankle motion by 60% and 65%, respectively (relative to the knee and ankle angles during mid-stance, which are not expected to change in late stance, based on healthy kinematics); and (2) the moderate amount of extension torque provided by the ECT was insufficient to fully reproduce healthy stair ascent behavior, implying that more torque is necessary to do so. However, given that the ECT provides about 30% of the extensive torque of the biological limb (for the subject’s mass), a 60–65% reduction in compensatory motion indicates that it may not be necessary to provide 100% of the torque of the biological limb to improve step-over stair ascent.

To contrast the compensatory strategy used with the ECT, the subject employed a different compensatory strategy with the MPK. During swing-phase on the prosthetic side, the subject abducted his thigh (70–100% of stride, Figure 6c) while simultaneously over-extending his intact side ankle (30–50% of stride, Figure 6d), which provided the necessary clearance to place the prosthetic foot on the next step. Once placed, the prosthetic knee was straight, which required the subject to forcefully adduct his prosthetic side thigh (0–30% of stride, Figure 6c) while over-extending his intact side knee and ankle (50–65% of stride, Figure 6b,d). These different compensatory strategies can be observed in the Supplementary Video. Of the three methods of stair ascent presented here, the subject indicated that the ECT with powered stance assistance was the most preferable (see the “Subjective Feedback” section), which may indicate that the least amount of compensatory effort was required in that case.

### Sit-to-stand and stand-to-sit transition

The ECT demonstrated similar performance to the MPK during stand-to-sit transitions and superior performance to the MPK during sit-to-stand transitions. Figure 7 shows a comparison between the prosthetic and intact sides while wearing an MPK and the ECT (with stance assistance) during sit-to-stand and stand-to-sit. These data show that providing an assistive knee torque during sit-to-stand reduced the ground reaction force (100 N/12% reduction in peak force) and knee power (11 J/10% reduction in total positive knee energy) on the subject’s intact side, while simultaneously increasing those metrics on the prosthetic side (125 N/80% increase in peak force; 25 J increase in total positive knee energy), which are similar results observed in Wolf et al. (2012).

During stand-to-sit, the expected outcome was that there would be no difference between the MPK and ECT, since it was hypothesized that each prosthesis provides the same turbulent damping resistance to motion. While there are no differences between most biomechanical metrics, the total energy dissipated on the intact side is lower for the MPK than for the ECT, which was an unexpected result. In evaluating the torque–speed behavior of the MPK in the stand-to-sit trials, the authors observed that the control system of the C-Leg 4 increases knee flexion resistance to a very high level before the subject begins sitting. This means that the two prostheses do not have the same constitutive behavior during stand-to-sit, which may be the source of the differences in experimental outcomes. Note that, given the variability in the stand-to-sit data, additional data are necessary to identify significant differences.

### Over-ground walking circuit

Figure 8 shows the results of the over-ground walking circuit, where the subject performed a variety of activities with the ECT. The figure shows both knee angle and controller state as a function of time to demonstrate the state-flow of the FSM within and between each activity and the corresponding knee motion. The figure reflects the following sequence of activities: the subject stood up from a chair, walked on level ground, up a ramp, made an outside turn, down stairs, level ground, made an inside turn, walked on level ground, walked backward, walked forward again, ascended stairs step-over-step, made an inside turn, walked down a ramp, walked on level ground, and sat down. No hesitation nor special movement was required between activities (as indicated in the corresponding Supplementary Video) suggesting that the FSM transition conditions provided for automatic transitions based upon how the user moved the prosthesis. To transition to down-slope or down-stairs requires the same user motion as required by the C-Leg to utilize stance yielding; to transition to up-stairs, the subject unloads the prosthesis with an extended knee while stepping up with his contralateral leg; to initiate powered sit-to-stand, the user need only begin extending his hip while loading the prosthesis. In addition to providing appropriate gait activity, the FSM permits all transitions between activities to be facilitated through natural user motion. This control structure therefore empowers the user to control the constitutive behavior of the prosthesis through movement.

### Subjective feedback

When combined with quantitative data, qualitative user feedback provides an important perspective on the assessment of novel design approaches. To give a lens to this new approach to prosthesis design and control, and to provide insight into future developments of this design approach, the study participant was given a follow-up survey to state his preferences for either the C-Leg or ECT knee in each of the experimental activities. The survey and its results are shown in Table 5. For each of the experimental activities, the subject was instructed to select one of the five options below that best describes his preference:

- Greatly prefer the C-Leg.
- Somewhat prefer the C-Leg.
- No difference in preference.
- Somewhat prefer the ECT knee.
- Greatly prefer the ECT knee.

**Table 5.** Post-study survey and interview responses

Q. no.	Which prosthesis do you prefer when …	Survey response
1	… walking at a *slow* *speed* on a flat treadmill?	*No difference* in preference
	*Interview response*: Swing assistance makes it easier to walk slowly because I do not have to work as hard to swing the leg. But it is uncomfortable to walk slowly, since I have to spend so much time standing on the prosthesis, so it is difficult to notice the difference that was being made by swing assistance.	
2	… walking at a *comfortable speed* on a flat treadmill?	*No difference* in preference
	*Interview response*: The ECT did a great job emulating the C-Leg; I had no problem walking with the ECT at these speeds. [The C-Leg and ECT] are very much the same when walking.	
3	… walking at a *fast speed* on a flat treadmill?	*Somewhat* prefer the *C-Leg*
	*Interview response*: The C-Leg was smoother than the ECT. When the knee extended fully at the end of swing, the ECT was hitting harder than I wanted. But the C-Leg did that more smoothly.	
4	… walking *up-slope* on a treadmill?	*Somewhat* prefer the *ECT knee*
	*Interview response*: I liked the swing-assistance for going up-slope. The foot seemed to go into the right place, because the foot extends out further. Like the foot placement was more appropriate for going up a slope. With the C-Leg, my [prosthetic] foot does not get as far out.	
5	… walking *down-slope* on a treadmill?	*Somewhat* prefer the *ECT knee*
	*Interview response*: The ECT had more resistance than the C-Leg. The C-Leg feels like it starts to collapse too soon. The ECT held me up better and I was walking smoother. My steps with the C-Leg end too soon.	
6	… walking *up-stairs*?	*Greatly* prefer the *ECT knee*
	*Interview response*: With the C-Leg, I have to pole-vault up the stairs. I have to put my leg out to the side and stiff leg it up there. That’s not how people climb stairs. With the ECT, I can actually walk up step over step. The motor really helps me climb stairs, to position the foot, with the whole process. I was actually getting pretty good at it. I think with experience and practice I could to it really smoothly. However, the prosthesis takes its time in swing. Getting the foot up there and placing it on the next step was a little slow. I’d like it to go faster than that. Also, it needs more power in stance, probably 50% more power.	
7	… walking *down-stairs*?	*Somewhat* prefer the *ECT knee*
	*Interview response*: I felt like I was in control of the ECT. With the ECT, I could go down-stairs without holding the handrail. I cannot do that with the C-Leg because it collapses too quickly, and I do not want to injure my knee. The ECT went downstairs smoothly. (Note that the subject was instructed to use a handrail during stair descent for safety reasons.)	
8	… *standing up* from a chair?	*No difference* in preference
	*Interview response*: I was able to stand up faster with the ECT, and the assistance does help a bit. But my [intact] leg has to do the bulk of the work, so the difference is not that much.	
9	… *sitting down* to a chair?	*No difference* in preference
	*Interview response*: The resistance does not matter as much when sitting down because my [intact] leg is doing most of the work. Resistance matters when going down stairs and slopes because I’m putting all my weight on the prosthesis. It might help a little bit, but it’s not enough to put even weight on both legs.	
10	Which prosthesis do you prefer *overall*?	*No difference* in preference
	*Interview response*: The ECT was superior when ascending/descending stairs and slopes. The C-Leg was a bit smoother on level-ground walking. And the C-Leg is quieter.	

After the survey, an interview was conducted where the subject was asked open-ended questions about his responses to the survey. For experimental activities where the subject preferred the C-Leg over the ECT knee, the subject was asked the following two questions:

- What about your experience with the C-Leg made it preferable during this activity?
- What about your experience with the ECT knee made it less preferable during this activity?

For experimental activities where the subject preferred the ECT knee over the C-Leg, the subject was asked the following two questions:

- What about your experience with the ECT knee made it preferable during this activity?
- What about your experience with the C-Leg made it less preferable during this activity?

For experimental activities where there was no preference for one over the other, the subject was asked the following question:

- Why was there no preference for either device?

## Discussion

### Enabling human–prosthesis interaction via low impedance

While passive prosthetic knees can only react, PKs can both act and react (i.e., they are capable of producing motion independent of user inputs), thus complicating the human–prosthesis interaction. The controlled interaction between the prosthesis and user therefore becomes substantially more important in PKs. In a low-impedance state, the prosthesis is generally more receptive to user effort, and therefore, it is both more controllable by a user, and more sensitive to user input, relative to a high-impedance state. Specifically, impedance is the ratio of (generalized) force to motion (i.e., velocity), meaning that a low impedance will result in increased motion for a given user force input. Therefore, a prosthetic knee capable of assuming a low-impedance state increases: (1) the control the user has over its motion, and (2) the sensitivity of the prosthesis to user input and intent. Strategic implementation of a low-impedance interaction has the potential to enhance the human–prosthesis interaction.

MPKs interact with users by providing an impedance that is both appropriate for the current action (e.g., high-impedance during stance-phase and a low impedance during swing-phase) and appropriate for detecting the user’s intent to act (e.g., low impedance in late-stance, prior to swing-phase). While PKs use sensing to infer user intent, their relatively higher output impedance reduces their ability to be as receptive to user input as are MPKs. There are several common actions taken by prosthesis users where, in the opinion of the authors, maintaining a low output impedance behavior such as proposed here, can greatly facilitate inferring intent from a user and providing physical coordination with him or her, including:

*Initiation of swing-phase* – A low output impedance in late-stance and throughout swing-phase reduces the effort needed by the user to initiate swing-phase and provides for a natural coordination of the user’s motion and prosthesis behavior

(since it is provided as a reaction to user motion).

*Ballistic swing* – A biomimetic reproduction of healthy swing-phase dynamics necessitates that the prosthetic knee provides a ballistic swing-phase motion. Additionally, because swing-phase is a net-dissipative knee behavior for most walking speeds, this behavior can be provided entirely by motor braking. The more movements that can be done passively, the lower the power requirements for locomotion.

*Non-ballistic swing* – Like ballistic swing, a low output impedance enables natural coordination of the intent to initiate a powered non-ballistic swing-phase during late stance. Additionally, a low-impedance non-ballistic swing reduces the actuator torque required to move the knee joint (due to reduced transmission dynamics, resulting in higher overall efficiency), provides a more natural disturbance rejection (due to reduced inertia and Coulomb friction that otherwise act as a low admittance to disturbances, including disturbances that result from swing-phase motion of the user’s residual thigh), and may reduce the back EMF when tracking high-velocity trajectories (due to the reduced transmission ratio, as demonstrated in this manuscript).

*Initiation of powered knee extension* – During the stance-phase, the intent to initiate generalized resistive and active behaviors can be communicated to the prosthesis via flexion or extension of the user’s hip musculature (because the stance leg is a closed kinematic chain, this motion is translated to the knee joint). While this intent to flex or extend the knee can be sensed as a torque in a high-impedance device, sensing with motion has the benefit of observing the user’s movement and reacting to it. Because stance-phase torques are high with relatively smaller accelerations, it is less important to have low actuator inertia, compared to swing-phase. However, it is important to have low stance-phase friction at low knee velocities, where the direction of motion inflects (i.e. stiction, motor cogging, and Coulomb friction must be low), which otherwise increases the effort required by the user to initiate knee flexion or extension.

*User acceptance of powered knees* – Prosthesis users are accustomed to interacting with passive devices. Designing PKs with passive dynamics akin to state-of-the-art passive prostheses has the potential to aid with accommodation and ultimate acceptance of PKs. In this case, the replication of MPK-like behavior on the ECT allowed the user to quickly adapt to the function of the new prosthesis.

### Using power to assist motion, rather than control motion

The proposed controller is premised on the idea that, whenever possible, the prosthesis should default to behavior that requires it to react to user inputs, rather than act in anticipation of user motion. While this behavior is inherent when the prosthesis is controlled passively like an MPK, the idea behind this approach can be extended to active behaviors as well. Power can be used to either control the motion of the knee, or to assist the motion of the knee by “pushing” it in the direction that the user is moving it. Utilizing power to do the latter allows the user to initiate motion before the prosthesis provides powered assistance to that motion. Two examples of power assistance are demonstrated in this article: (1) During slow walking, inertial forces are insufficient to produce enough knee flexion during swing-phase, even in low-impedance knee prostheses, which leads to compensatory motion to enable ground clearance of the foot. In these cases, power can be used to “push” the knee toward flexion during late-stance and swing-flexion phases. This preserves the inertial coupling of swing-phase without compromising ground clearance, as the increased knee flexion can potentially reduce the compensatory motion required by the user to avoid catching the toe of the prosthesis during slow walking, since most prosthetic feet cannot actively dorsiflex like the biological ankle does during swing-phase. (2) During activities that involve substantial stance knee-extension, the stance leg is a closed kinematic chain, which allows the prosthesis user to control knee joint movement via movement of the hip joint. While possible for the user to extend the knee joint using hip musculature alone (e.g., Lee and Goldfarb, 2021), this motion can be achieved with less user effort by providing extensive power at the knee. Just as the biological hip and knee work synergistically to extend the leg, the prosthetic knee is able to follow motion and force cues from the residual hip and provide synchronous assistive knee torque. In this manner, the user does not react to the knee’s control of motion, but rather works with it to extend the leg, similar to the manner in which an electric bicycle coordinates its power delivery with the user’s power input. Although a knee prosthesis is often thought of as a replacement of a joint, as opposed to augmentation of a joint, in the case of stance-knee extension during stair ascent, the knee is essentially in parallel with the hip joint; as such, the knee in stance might better be thought of as a joint that augments hip effort to extend the whole leg, rather than as a joint that acts in series with it.

Finally, it should be noted regarding all discussion points that the experience reflected in this discussion is based primarily on testing with a single individual with transfemoral amputation. The authors have tried to convey insights gained from experience with this approach to powered prosthesis implementation; however, the extent to which these perspectives can be generalized is of course quite limited.

### Hardware limitations

The back-drivable lead screw in the ECT’s drivetrain has design tradeoffs that benefited passive behavior but degraded active performance. The amount of friction in the leadscrew is torque-dependent. As such, at low actuator torques (e.g., ballistic swing), the amount of friction is very low, while at high actuators torques, the friction is considerably higher. This load-dependent friction creates a benefit for both resistive stance and ballistic swing by amplifying the resistive motor torque by a factor of two, which increases the range of controllable impedances in the low-gear and protects the planetary gear transmission from overload in the high-gear. However, these benefits come at the cost of decreased efficiency in the forward drive, increasing the electrical energy cost of non-ballistic swing and severely decreasing the maximum stance-phase assistive torque to levels that compromise performance in stair ascent and stand-to-sit activities. For a future version of this design, the designer should consider use of power transmission components that have a better balance of forward-drive and back-drive efficiencies, which will change the ratio of maximum forward-drive and back-drive torques. While necessary to provide high resistive torques in stance (from embedded system data and a model of actuator friction, we calculated the knee torque to be up to 80 N-m during stair descent, which is expected from previous studies [Schmalz et al., 2007]), it may not be necessary to provide a biomimetic magnitude of knee extension torque during stair ascent or sit-to-stand to provide benefit to powered knee extension. As demonstrated during stair ascent and sit-to-stand, an assistive torque approximately 30% of the biological limb’s for the subject’s mass was sufficient to increase prosthetic knee power and ground reaction force, reduce intact limb knee power and ground reaction force, and reduce intact limb compensatory action (e.g., rapid flexion and extension of the intact knee and ankle during late-stance of stair ascent). This is not surprising considering that MPK users average about 75% of the biological knee torque with their prosthesis during stair descent, and transtibial amputees use approximately 25% of the biological knee torque with their prosthetic side knee during stair ascent (Schmalz et al., 2007), indicating that they may be unwilling to load the prosthesis to biomimetic levels during stair ambulation. Therefore, it may be unnecessary and perhaps undesirable to provide 100% of the biological knee’s extension torque during stair ascent and sit-to-stand.

## Conclusion

This article describes a control approach for a powered knee prosthesis that provides a nominally strictly passive functionality, and layers powered assistance onto the nominally passive functionality when appropriate. The control system was implemented on a novel powered prosthetic knee that enables low-impedance behaviors associated with ballistic swing, and high-torque behaviors associated with passive and powered stance, by employing both passive and active motor control in combination with an electronically switchable two-speed transmission. Experiments comparing the control approach against a strictly passive MPK demonstrate the ability of the proposed approach to provide essentially identical passive behaviors to the MPK, while also providing powered behaviors to aid stair ascent, slow walking, and sit-to-stand transitions.

## Supporting information

Culver et al. supplementary material Culver et al. supplementary material

## Data Availability

The data that support the findings of this study are available on request from the corresponding author, S.C.C.

## Supplementary material

The supplementary material for this article can be found at https://doi.org/10.1017/wtc.2023.14.
